# Validation of the Critical-Care Pain Observation Tool-Neuro in brain-injured adults in the intensive care unit: a prospective cohort study

**DOI:** 10.1186/s13054-021-03561-1

**Published:** 2021-04-13

**Authors:** Céline Gélinas, Mélanie Bérubé, Kathleen A. Puntillo, Madalina Boitor, Melissa Richard-Lalonde, Francis Bernard, Virginie Williams, Aaron M. Joffe, Craig Steiner, Rebekah Marsh, Louise Rose, Craig M. Dale, Darina M. Tsoller, Manon Choinière, David L. Streiner

**Affiliations:** 1 Ingram School of Nursing, McGill University, 680 Sherbrooke West St., Suite 1800, Montreal, QC H3A 2M7 Canada; 2 Centre for Nursing Research and Lady Davis Institute, Jewish General Hospital, CIUSSS West-Central Montreal, 3755 Côte-Sainte-Catherine Road, Montreal, QC H3T 1E2 Canada; 3 Faculty of Nursing, Université Laval, 1050 Avenue de la Médecine, Room 3486, Quebec City, QC G1V 0A6 Canada; 4 Population Health and Optimal Health Practices Research Unit, Trauma-Emergency-Critical Care Medicine, Centre de Recherche du CHU de Québec – Université Laval, 1401, 18e rue, Room Z-243, Quebec City, QC G1J 1Z4 Canada; 5 Physiological Nursing, University of California San Francisco, 2 Koret Way, San Francisco, CA 94143 USA; 6 Faculty of Dentistry, McGill University, 3640 University St., Montreal, QC H3A 0C7 Canada; 7 Équipe de Recherche en Soins Intensifs (ERESI), Research centre, Centre Intégré Universitaire de Santé et de Services Sociaux du Nord-de-l’île-de-Montréal, Hôpital du Sacré-Coeur-de-Montréal, 5400 boulevard Gouin Ouest, K-3000, Montreal, QC H4J 1C4 Canada; 8 Department of Medicine, Université de Montréal, Succursale Centre-Ville, C.P. 6128, Montreal, QC H3C 3J7 Canada; 9 School of Medicine, University of Washington, 1959 NE Pacific St, Seattle, WA 98195 USA; 10 Harborview Medical Center, University of Washington Medicine, 325 9th Avenue, Seattle, WA 98104 USA; 11 Florence Nightingale Faculty of Nursing, Midwifery and Palliative Care, King’s College London, 57 Waterloo Rd, London, SE1 8WA UK; 12 Lawrence S. Bloomberg Faculty of Nursing, University of Toronto, 155 College Street, Suite 130, Toronto, ON M5T 1P8 Canada; 13 Tory Trauma Program, Sunnybrook Health Sciences Centre, 2075 Bayview Avenue, Toronto, M4N 3M5 Canada; 14 Department of Anesthesiology and Pain Medicine, Faculty of Medicine, Université de Montréal, Succursale Centre-Ville, C.P. 6128, Montreal, QC H3C 3J7 Canada; 15 Research Center, Centre Hospitalier de l’Université de Montréal, Saint Antoine Building, Room S01-126, 850 Saint Denis St, Montreal, QC H2X 0A9 Canada; 16 Department of Psychiatry and Behavioural Neurosciences, McMaster University, St. Joseph’s Healthcare, 100 West 5th Street, Box 585, Hamilton, ON L8N 3K7 Canada

**Keywords:** Validation, Pain, Assessment, Brain injury, Critical care

## Abstract

**Background:**

Pain assessment in brain-injured patients in the intensive care unit (ICU) is challenging and existing scales may not be representative of behavioral reactions expressed by this specific group. This study aimed to validate the French-Canadian and English revised versions of the Critical-Care Pain Observation Tool (CPOT-Neuro) for brain-injured ICU patients.

**Methods:**

A prospective cohort study was conducted in three Canadian and one American sites. Patients with a traumatic or a non-traumatic brain injury were assessed with the CPOT-Neuro by trained raters (i.e., research staff and ICU nurses) before, during, and after nociceptive procedures (i.e., turning and other) and non-nociceptive procedures (i.e., non-invasive blood pressure, soft touch). Patients who were conscious and delirium-free were asked to provide their self-report of pain intensity (0–10). A first data set was completed for all participants (*n* = 226), and a second data set (*n* = 87) was obtained when a change in the level of consciousness (LOC) was observed after study enrollment. Three LOC groups were included: (a) unconscious (Glasgow Coma Scale or GCS 4–8); (b) altered LOC (GCS 9–12); and (c) conscious (GCS 13–15).

**Results:**

Higher CPOT-Neuro scores were found during nociceptive procedures compared to rest and non-nociceptive procedures in both data sets (*p* < 0.001). CPOT-Neuro scores were not different across LOC groups. Moderate correlations between CPOT-Neuro and self-reported pain intensity scores were found at rest and during nociceptive procedures (Spearman rho > 0.40 and > 0.60, respectively). CPOT-Neuro cut-off scores ≥ 2 and ≥ 3 were found to adequately classify mild to severe self-reported pain ≥ 1 and moderate to severe self-reported pain ≥ 5, respectively. Interrater reliability of raters’ CPOT-Neuro scores was supported with intraclass correlation coefficients > 0.69.

**Conclusions:**

The CPOT-Neuro was found to be valid in this multi-site sample of brain-injured ICU patients at various LOC. Implementation studies are necessary to evaluate the tool’s performance in clinical practice.

**Supplementary Information:**

The online version contains supplementary material available at 10.1186/s13054-021-03561-1.

## Background

Validation of behavioral pain scales in critically ill brain-injured patients in the intensive care unit (ICU) was identified as an area in need of future research in the 2013 clinical practice guidelines of the Society of Critical-Care Medicine (SCCM [1]). Since then, several studies have tested the two recommended scales, i.e., the Behavioral Pain Scale (BPS: [2]) and the Critical-Care Pain Observation Tool (CPOT: [3]) for pain assessment purposes in this specific ICU patient group; six studies used the BPS [4–9], six the CPOT [10–15], and one used both tools [16]. The BPS and the CPOT were validated in 193 and 690 brain-injured ICU patients, respectively, with more than half being mechanically ventilated. Higher behavioral scores during nociceptive procedures (e.g., turning, endotracheal suctioning) compared to rest or non-nociceptive procedures (e.g., eye care, non-invasive blood pressure [NIBP] with cuff inflation or soft touch) were consistently reported in all studies.

Despite the ability of the BPS and CPOT to discriminate between nociceptive and non-nociceptive procedures, several issues were identified at the item level. A lower effect size for responsiveness between rest and nociceptive procedures was found for the facial expression item compared to the upper limbs item of the BPS [9]. Also, grimace and muscle rigidity using the CPOT [11] were not frequently observed. However, grimace was the best predictor of self-reported pain intensity in brain-injured ICU patients who were conscious and able to communicate in a reliable manner, i.e., not delirious [17]. Behaviors not included in original versions of the scales such as orbit tightening, eye weeping (tearing), and face flushing were described in this patient population [17–19]. Low levels of consciousness (LOC) or high sedation levels, often present in brain-injured ICU patients, were associated with low frequency of behaviors indicative of pain [17–19] and low behavioral scale scores [8, 9, 12, 20]. Adaptation of the content of existing scales for brain-injured ICU patients could enhance their applicability and ability to accurately detect pain in this vulnerable population. Although other tools are available for brain-injured patients with disorders of consciousness such as the Nociception Coma Scale [21] and its revised version [22], these were not developed for use in the ICU context and are not applicable to mechanically ventilated patients [23].

The purpose of this study was to validate the use of the French-Canadian and English revised versions of the CPOT for brain-injured ICU patients: the CPOT-Neuro. Specific objectives were to examine the:

1. Association between CPOT-Neuro scores and the reference standard measure of pain (patient self-reporting), and the ability of CPOT-Neuro to detect self-reported pain in brain-injured ICU patients (criterion validation);
2. Ability of CPOT-Neuro scores to discriminate between non-nociceptive and nociceptive procedures, and when feasible, before and after opioid administration (discriminative validation);
3. Agreement of CPOT-Neuro scores between trained research staff and ICU nurses (interrater reliability)

## Methods

### Design

A prospective cohort with repeated-measures design was used for this multicenter validation study. This design allowed the testing of the reliability and the validity of the French-Canadian and English versions of the CPOT-Neuro with the collection of data across several procedures and time points.

### Settings

This study was conducted in four neurotraumatology ICU settings across Canada (two sites from Montreal, Quebec and one in Toronto, Ontario) and the USA (one site from Seattle, Washington). One of these settings was a French-speaking one (Montreal, Canada), one was bilingual (Montreal, Canada), and two were English-speaking settings (Toronto, Canada and USA). These ICU facilities had similar capacity (22–30 beds) admitting 1000–1500 patients annually. Individualized prescriptions were used for pain management in all sites.

### Sample

We sought a heterogeneous population of brain-injured ICU patients as we aimed to validate the CPOT-Neuro to be generalizable for a diverse brain-injured population, rather than specific to patients with a single diagnosis. Accordingly, patients meeting the following inclusion criteria were eligible: (1) 18 years and older; (2) admitted for brain injury to the ICU for less than 4 weeks (e.g., traumatic brain injury [TBI] with or without other trauma, ischemic or hemorrhagic stroke including cerebral aneurysm, cerebral tumor, or brain injury from other causes); and (3) had a score ≥ 4 on the Glasgow Coma Scale (GCS: [24]). To reduce potentially confounding factors and allow for the observation of behavioral reactions, patients were excluded if they: (1) sustained a spinal cord injury affecting motor activity of the four limbs; (2) had cognitive deficits or psychiatric conditions (e.g., psychosis, suicidal ideation) prior to the brain injury; (3) were previously diagnosed with epilepsy, (4) were receiving neuromuscular blocking agents; (5) had a score of − 5 (unarousable) on the Richmond Agitation Sedation Scale (RASS) [25]; and (6) had suspected brain death. According to the regulations in the provinces of Quebec and Ontario, informed written consent form was obtained from all participants (patients or their representative, in case of sudden incapability) of the three Canadian ICU settings. Because this was an observational and non-interventional study, informed written consent was not required according to regulations in the state of Washington. Recruitment and data collection occurred from June 2015 through December 2016.

### Procedures

Data collection was performed before and during non-nociceptive (NIBP, soft touch) and nociceptive (turning and others outlined in Table 1) procedures as well as before and after opioid administration. NIBP was measured with cuff inflation (equipment at the bedside) and was used as a non-nociceptive procedure as it was found to be painless in ICU patients including those with a brain injury [10, 15, 26]. NIBP and all nociceptive procedures were part of ICU standard care. Only soft touch was added as a non-nociceptive procedure during which research staff, an ICU nurse or a family member touched the patient’s forearm for one minute [27]. Soft touch was also found to be painless in brain-injured ICU patients [11, 13]. These procedures allowed evaluation of the ability of the CPOT-Neuro to discriminate between procedures likely to be painful or not (discriminative validation). Each patient had six-to-ten assessment time points (three to five procedures per patient for two assessment time points per procedure) per data set. All patients had at least one data set when initially enrolled in the study, and some of them had two data sets if their LOC changed during their ICU stay after study enrollment. LOC was evaluated according to their Glasgow Coma Scale (GCS) score: unconscious with GCS ≤ 8, altered LOC with GCS from 9 to 12, and conscious with GCS from 13 to 15 [24].

**Table 1:** Frequency of nociceptive procedures observed in the ICU

Nociceptive procedures	1st data set	2nd data set^b^
	Frequency (*n*)	Frequency (*n*)
Turning	211	81
Endotracheal suctioning	29	7
Mouth suctioning/care	16	5
Repositioning	14	1
Mobilization/physiotherapy	9	1
Parenteral injection/IV insertion	9	8
Blood draw	8	0
Dressing change	7	3
Finger prick	5	0
Arterial/central line removal	4	0
Drain removal	3	1
Nasogastric tube/Dobhoff insertion	3	0
Endotracheal tube removal	2	0
Other common ICU procedures^a^	9	1
Total	329	108

^a^Examples: Codman catheter insertion, manipulation of affected limb, collar care

^b^A second data collection was conducted with participants who experienced a change in their level of consciousness during their participation in this study

Patients were assessed for pain with the CPOT-Neuro by trained research staff. Whenever possible for turning and other nociceptive procedures, an available ICU nurse trained for the study was asked to independently complete the CPOT-Neuro to determine interrater reliability. Patients were assessed during one-minute periods or for the duration of the nociceptive procedure, and raters provided a score on each item of the CPOT-Neuro. After the completion of the tool, the research staff asked conscious and non-delirious patients to report the presence/absence of pain verbally (yes/no) or using signs (e.g., head nodding), and to rate their pain intensity using the 0–10 Faces Pain Thermometer (FPT) visual format [28]. The patient’s self-report is considered the reference standard measure in the field of pain and was used to establish criterion validation of the CPOT-Neuro.

### Instruments

#### CPOT-Neuro

The CPOT-Neuro is an adaptation of the original CPOT. The original tool [3] includes five behavioral items: (a) facial expression, (b) body movements, (c) compliance with ventilator (mechanically ventilated patients) or (d) vocalization (non-intubated patients), and (e) muscle tension. Each item is scored from 0 to 2 for a possible total score from 0 to 8. A higher score reflects more intense behavioral reactions, and a cut-off score ≥  2 indicates the presence of pain [10, 11, 23, 29].

In the adaptation process, each item and score of the original CPOT were reviewed in accordance with observational data of pain-related behaviors in brain-injured ICU patients [17, 18, 30]. Evaluations of the behaviors’ relevance for pain assessment in this population were also made by 61 ICU nurses, 13 physicians, and 3 physiotherapists [31].

Modifications were made to all items of the tool. Scores of 0 remained unchanged for facial expression, body movements, muscle tension, compliance with the ventilator, and vocalization. For the facial expression item, the score of 1 was modified to only include brow lowering. Brow lowering was identified as a frequent reaction to a painful procedure in brain-injured ICU patients no matter their LOC [17, 18], and considered relevant by ICU clinicians [31]. A score of 2 was modified to include at least two contractions in the patient’s upper face (e.g., brow lowering + eye tightening) or grimace. Grimace is the strongest predictor of pain intensity in this patient group [17] and rated as highly relevant by ICU clinicians [31]. In regard to body movements, scores of 1 and 2 were also operationalized a bit differently. A score of 1 was modified to include non-purposeful movements such as cautious movements or limb flexion. A score of 2 was related to protection or purposeful movements such as trying to reach or touching the pain site which was rated as highly relevant by ICU clinicians [31]. These descriptions of body movements better reflect what was observed in brain-injured ICU patients [17]. In the ventilator compliance item, only the description of score of 1 was modified for activation of alarms. Coughing was removed as it was rated as irrelevant by ICU clinicians [31]. For vocalization, verbal complaints of pain were added in the score of 2 as it was considered relevant by ICU clinicians for brain-injured patients who are conscious or with an altered LOC [31]. The score of 2 (very tense) for muscle tension was removed as it can be confounded with spasticity as a consequence of brain injury as highlighted by ICU clinicians [31]. Autonomic responses related to tearing and face flushing were newly described in brain-injured ICU patients [17, 19]. Autonomic responses were also rated as relevant by clinicians for brain-injured ICU patients who are conscious or with an altered LOC [31]. A score of 1 was assigned in the presence of at least one of these autonomic responses. The total score of CPOT-Neuro may vary from 0 to 8 and remains consistent with the original CPOT total score.

The CPOT-Neuro was initially adapted in French-Canadian from the original CPOT French-Canadian version [32] and then translated into English using a forward–backward translation method. Both the French-Canadian and the English versions of the CPOT-Neuro were validated simultaneously in this study. Description of the CPOT-Neuro can be found in Additional file 1.

#### Raters and CPOT-Neuro Training

The raters included 11 research staff and 36 ICU nurses. Research staff included 2 nursing researchers, 2 clinical research coordinators, 6 nursing research trainees, and one medical student. ICU nurses were mainly female (89%), held a college (33%) or a university degree (66%), and had an experience in the ICU ranging from 2 to 34 years (median = 8.00 [IQR = 5.25–16.75]). In one Canadian ICU, only research staff completed CPOT-Neuro assessments. In other ICUs, both research staff and ICU nurses completed CPOT-Neuro assessments.

The raters were trained by the PI who is also the author of the CPOT/CPOT-Neuro (CG) employing a standardized training session based on the one used for the original CPOT [33]. The training session lasted 45 min during which each item and scoring of the CPOT-Neuro were explained in detail with the support of illustrations and pictures. Briefly, training sessions were delivered in small groups or individually. Three patient videos were also viewed to practice scoring with the tool and to discuss scores within the group or with individual raters. At the end of the training session, raters were asked to view three other patient videos and to provide their written scores. It was expected that raters would use the CPOT-Neuro consistently resulting in a total score difference of no more than one point which was considered acceptable in previous training with the CPOT [34]. When a difference of two points or more was found, the CPOT-Neuro scoring methods were clarified before moving to the next patient video. More than 73% of raters at each site had appropriate scores for all three patient videos, and fewer than 25% had difference in scores ≥ 2 for a maximum of two patient videos [35]. An overall Intraclass Correlation Coefficient (ICC) of 0.85 (95% CI [0.59–1.00]) with all raters’ scores for the three videos was reached. Further details on the training are described in another paper [35].

#### Participant’s self-report

At each time point after the completion of the CPOT-Neuro, conscious patients who were able to self-report their pain and not delirious (see *Screening of delirium*) were asked the question “Do you have pain?”, and answer “yes or no” verbally or with other signs (e.g., head nodding). Then, they were asked to rate their pain level with the 0–10 Faces Pain Thermometer (FPT) visual format. The FPT has been developed and tested in critically ill adults by the PI [28], and used in studies with brain-injured ICU patients [10, 11, 17, 18, 28]. It consists of a thermometer graded from 0 (no pain) to 10 (worst possible pain), including six faces adapted from Prkachin’s work [36]. The scale demonstrated good convergent (*r* = 0.80–0.86 with a descriptive rating scale: [28]) and discriminative validation when comparing scores at rest, during nociceptive and non-nociceptive procedures [10, 11, 26, 28, 30, 37].

#### Socio-demographic and clinical variables

The following were collected for each participant: demographic information (sex and age) and clinical data from the patient’s medical chart such as diagnosis, severity of illness (APACHE II score: [38]), neuroanatomical location of lesion (diffuse, frontal, parietotemporal, occipital, subcortical), level of consciousness (GCS score or adapted version) [24, 39], level of sedation (RASS score: [25]), and administration of analgesic/sedative agents (i.e., IV infusions, and boluses administered within an hour prior to procedures).

#### Screening of delirium

Prior to data collection, delirium was screened in all conscious patients who were able to communicate verbally or use signs (e.g., head nodding). Delirium can compromise the reliability of self-reports [23]. Trained research staff used either the CAM-ICU [40] or the ICDSC (Intensive Care Delirium Screening Checklist: [41]) whichever was standard practice in the ICU setting to screen patients for delirium. The CAM-ICU and the ICDSC were found to be the most reliable and valid delirium assessment tools for use in critically ill adults based on a recent systematic review [42].

### Data analysis

The sample size for this study was calculated based on the requirements for the evaluation of criterion validation using the reference standard measure of pain (patient self-reporting), and to include brain-injured ICU patients at various LOC which is representative of this population. Using MedCalc and G*Power for sample size estimation, a minimal sample size of 52 conscious patients able to self-report their pain was needed to obtain a moderate correlation of 0.50 as found in previous CPOT validation studies [3, 10, 11, 26, 43, 44] with 90% power and a significance level of 0.01 based on Bonferroni correction for multiple tests. A sample of 50 patients was required for a Receiver Operating Characteristic (ROC) curve analysis with an AUC of 0.80 and a ratio of negative/positive cases of 0.6 during procedural pain [11, 29], with 90% power and a significance level of 0.01. Wilcoxon Signed Ranks test with a minimum sample of 50 brain-injured ICU patients in each group of the three LOC with an effect size of 0.50 and a significance level of 0.01 would allow a power of 85%.

SPSS software (version 24.0) was used for data analysis. Descriptive statistics were calculated for socio-demographic and clinical data. Nonparametric tests were used given that variables were not normally distributed as supported by Kolmogorov–Smirnov and Shapiro–Wilk tests with *p* > 0.05 at all assessment time points, and skewness and kurtosis indices >  ± 2 [45] for most time points except during turning and other procedures. Kruskal-Wallis and Mann–Whitney *U* tests were performed to compare CPOT-Neuro scores across analgesia/sedation regimen, medical diagnosis, and sedation level groups. Criterion validation was estimated using the Spearman’s rho correlation coefficient between the patients’ self-report of pain intensity and CPOT-Neuro scores. The Receiver Operating Characteristic (ROC) curve analysis was used to evaluate the ability of the CPOT-Neuro to classify patients who self-reported the presence versus absence of pain and different pain intensity levels (i.e., ≥ 1 for all levels of pain and ≥ 5 for moderate to severe levels of pain), and to determine the best cut-off score at these pain intensity levels. Discriminative validation of CPOT-Neuro scores across time points and procedures was examined using the Wilcoxon Signed Ranks test. Finally, interrater reliability between the research staff and ICU nurses was examined using the Intraclass Correlation Coefficient (ICC) (two-way mixed model). Missing data were not replaced. Data analyses were performed by a PhD-prepared health professional who was not involved in data collection.

## Results

### Sample description

**Fig. 1:**
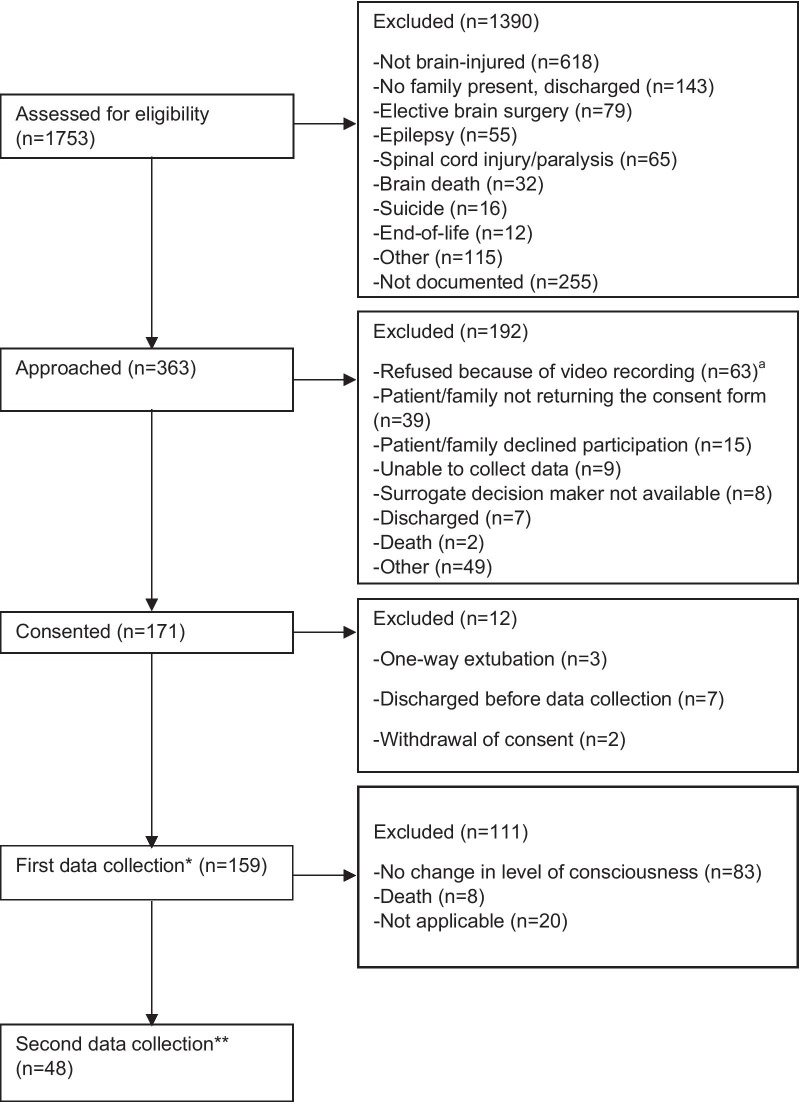
Participant flow diagram—Canadian sites. ^a^Patients were video recorded in Montreal sites only. *67 from the American site for a total of 226 participants in the first data set. **39 from the American site for a total of 87 participants in the second data set

A total of 226 patients participated and had a first data set collected. Of these, 87 had a second data set collected when they experienced a change in their LOC during their ICU stay (see Flow Diagram in Fig. 1). In both data sets, patients had a median age > 50 years, the majority were male (> 65%), White Caucasian (> 80%), and admitted to the ICU following a TBI (> 55%) with a brain injury mostly located in the frontal lobe (Table 2). RASS sedation levels indicated that patients were generally drowsy (− 1) or sedated. Almost half were mechanically ventilated.

**Table 2:** Socio-demographic and medical data of brain-injured ICU patients at each data set

	1st data set (*n* = 226)	2nd data set (*n* = 87)
Age (years)		
Median	58	53
Interquartile range (IQR)	39.5–75	37–67
Sex: *n* (%)		
Male	154 (68%)	57 (66%)
Female	72 (32%)	30 (34%)
Diagnosis: *n* (%)		
Traumatic brain injury (TBI)	134 (59%)	48 (55%)
Neuro-medical^a^	92 (41%)	39 (45%)
Location of brain injury in	*n* = 93	*n* = 34
TBI Patients: *n*		
Frontal	78	33
Parietal	40	11
Temporal	46	15
Occipital	19	10
Missing	41	14
Mechanically ventilated: *n* (%)	101 (45%)	46 (53%)
APACHE^a^ score		
Median	16	18
Range	0–35	6–35
Level of Consciousness: *n* (%)		
Unconscious (GCS^b^ 4–8)	36 (16%)	14 (16%)
Altered (GCS 9–12)	63 (28%)	27 (31%)
Conscious (GCS 13–15)	127 (56%)	46 (53%)
RASS^c^		
Median	− 1	− 1
Range	− 4 to + 3	− 4 to + 3
Missing data *n* (%)	24	–
Awake (RASS = 0)	73 (32%)	20 (23%)
Sedated (RASS = − 1 to − 4)	104 (46%)	52 (60%)
Agitated (RASS = + 1 to + 3)	25 (11%)	15 (17%)
CAM-ICU^d^: *n* (%)		
Negative	74 (33%)	18 (21%)
Positive	20 (9%)	10 (11%)
Not Measurable	132 (58%)	59 (68%)

Injury could be located in more than one area

Neuro-medical diagnoses include ischemic and hemorrhagic stroke, cerebral aneurysm and tumor, and other non-traumatic brain injury

^a^APACHE: Acute Physiology And Chronic Health Evaluation

^b^GCS: Glasgow Coma Scale

^c^RASS: Richmond Agitation-Sedation Scale

^d^CAM-ICU: Confusion Assessment Method for the Intensive Care Unit

Regarding analgesia and sedation for the first data set, more than half of patients did not receive analgesia/sedation before the nociceptive procedures (turning: 54%, other procedures: 53%), some received continuous analgesia and/or sedation (turning: 41%, other procedures: 42%), and only 5% and 4% of patients had a bolus of analgesia/sedation within one hour before turning and other procedures, respectively. Total CPOT-Neuro scores during turning and other nociceptive procedures were not significantly different across analgesia/sedation regimen (Kruskal Wallis test = 0.35 and 1.91, *p* = 0.950 and 0.591) and medical diagnosis (TBI versus neuro-medical; Mann–Whitney *U* test = 4859.00 and 1501.50, *p* = 0.268 and 0.914). Similar results were obtained for the second data set, where 54% and 44% of patients did not receive analgesia/sedation, 37% and 40% received continuous analgesia and/or sedation, and only 9% and 15% had a bolus of analgesia within one hour before turning and other procedures, respectively. In addition, total CPOT-Neuro scores during turning and other nociceptive procedures were not different across analgesia/sedation regimen (Kruskal Wallis test = 0.87 and 6.25, *p* = 0.832 and 0.100) and medical diagnosis (Mann–Whitney *U* test = 809.50 and 36.50, *p* = 0.943 and 0.502).

A total of 95 conscious patients could self-report pain during turning either during the first or the second data set, 18 screened positive for delirium as per the CAM-ICU or ICDSC and were excluded from criterion validation analyses. Similarly, 39 could self-report during other nociceptive procedures and nine were excluded for being positive on delirium screening. Therefore, criterion validation analyses included the self-report of 77 patients during turning, and 30 during other nociceptive procedures. All self-reports were independent data.

### Description of CPOT-Neuro scores

During the first data set, total CPOT-Neuro scores were low (median = 0) when patients were at rest, post-administration of an opioid, and during non-nociceptive procedures such as NIBP and soft touch (Table 3). At these time points, patients expressed mainly neutral behaviors such as relaxed facial expression, absence of autonomic responses and body movements, relaxed muscles and compliance with the ventilator or normal vocalization. Higher total CPOT-Neuro scores were observed prior to opioid administration, during turning and other nociceptive procedures. Similar results were obtained for the sub-group of patients with a second data set (Table 4).

**Table 3:** Frequency (%) of behavioral items and descriptive statistics of CPOT-Neuro scores during the first data set

Items	Procedures									
	Turning		Other nociceptive procedures		Opioid administration		Non-invasive blood pressure		Soft touch	
	Pre (*n* = 211)	During (*n* = 211)	Pre (*n* = 117)	During (*n* = 118)	Pre (*n* = 35)	Post (*n* = 35)	Pre (*n* = 205)	During (*n* = 206)	Pre (*n* = 219)	During (*n* = 219)
Facial expression										
Relaxed	183 (87)	80 (38)	101 (86)	41 (35)	25 (71)	32 (91)	177 (86)	164 (80)	184 (84)	186 (85)
Brow lowering	16 (8)	52 (25)	10 (9)	19 (16)	1 (3)	2 (6)	16 (8)	27 (13)	22 (10)	23 (11)
Upper face or Grimacing	12 (6)	79 (37)	6 (5)	58 (49)	9 (26)	1 (3)	12 (6)	15 (7)	13 (6)	10 (4)
Autonomic responses (tearing, face flushing)										
Absence	199 (94)	155 (73)	113 (97)	88 (75)	32 (91)	34 (97)	201 (98)	201 (98)	212 (97)	213 (97)
Presence	12 (6)	56 (27)	4 (3)	30 (25)	3 (9)	1 (3)	4 (2)	5 (2)	7 (3)	6 (3)
Body movements										
Absence of movements	168 (80)	106 (50)	96 (82)	48 (41)	17 (49)	25 (71)	172 (84)	163 (79)	179 (82)	174 (79)
Non-purposeful movements	29 (14)	64 (30)	17 (15)	40 (34)	12 (34)	6 (17)	24 (12)	29 (14)	25 (11)	33 (15)
Purposeful movements	14 (6)	41 (20)	4 (3)	30 (25)	6 (17)	4 (12)	9 (4)	14 (7)	15 (7)	12 (6)
Muscle tension										
Relaxed	189 (90)	144 (68)	105 (90)	80 (68)	22 (63)	32 (91)	191 (93)	186 (90)	201 (92)	201 (92)
Tense	22 (10)	67 (32)	12 (10)	38 (32)	13 (37)	3 (9)	14 (7)	20 (10)	18 (8)	18 (8)
*Mechanically ventilated*										
Compliance with ventilator										
Tolerating ventilator	94 (45)	50 (24)	60 (51)	38 (32)	17 (49)	22 (63)	87 (42)	85 (41)	96 (44)	99 (45)
Activating alarms	2 (1)	43 (20)	–	18 (15)	5 (14)	–	−	2 (1)	–	−
Fighting ventilator	–	3 (1)	–	3 (3)	–		–	–	–	–
*Not mechanically ventilated*										
Vocalization										
Normal tone/no sound	109 (52)	74 (35)	52 (44)	33 (30)	8 (23)	10 (29)	111 (54)	109 (53)	116 (53)	115 (53)
Sighing/moaning	5 (2)	20 (9)	3 (3)	15 (13)	3 (9)	3 (9)	5 (2)	6 (3)	4 (2)	4 (2)
Verbal complaints	1 (0.5)	21 (10)	2 (2)	11 (9)	2 (6)	–	2 (1)	4 (2)	3 (1)	1 (0.5)
CPOT-Neuro scores										
Median	0	3	0	3	1	0	0	0	0	0
Range	0–6	0–8	0–6	0–8	0–7	0–3	0–6	0–5	0–7	0–6
IQR	0–1	1–4	0–1	1–5	0–4	0–1	0–1	0–1	0–1	0–1
Mean	0.66	2.79	0.59	3.08	2.03	0.71	0.53	0.75	0.63	0.59
SD	1.11	1.98	1.15	2.05	2.28	0.96	1.03	1.11	1.17	1.07

*IQR* interquartile range, *SD* standard deviation

**Table 4:** Frequency (%) of behavioral items and descriptive statistics of CPOT-Neuro scores during the second data set

Items	Procedures									
	Turning		Other nociceptive procedures		Opioid administration		Non-invasive blood pressure		Soft touch	
	Pre (*n* = 81)	During (*n* = 81)	Pre (*n* = 27)	During (*n* = 27)	Pre (*n* = 11)	Post (*n* = 11)	Pre (*n* = 38)	During (*n* = 38)	Pre (*n* = 46)	During (*n* = 46)
Facial expression										
Relaxed	68 (84)	32 (40)	21 (78)	9 (33)	8 (73)	9 (82)	30 (79)	27 (71)	36 (78)	34 (74)
Brow lowering	12 (15)	20 (25)	5 (19)	6 (13)	2 (18)	2 (18)	6 (16)	3 (8)	6 (13)	8 (17)
Upper face or Grimacing	1 (1)	29 (35)	1 (4)	12 (44)	1 (9)	–	2 (5)	8 (11)	4 (9)	4 (9)
Autonomic responses (tearing, face flushing)										
Absence	77 (95)	60 (74)	26 (96)	20 (74)	10 (91)	11 (100)	37 (97)	37 (97)	45 (98)	46 (100)
Presence	4 (5)	21 (26)	1 (4)	7 (16)	1 (9)	–	1 (3)	1 (3)	1 (2)	−
Body movements										
Absence of movements	66 (81)	37 (46)	22 (81)	15 (56)	6 (55)	10 (91)	29 (76)	25 (66)	33 (72)	33 (72)
Non-purposeful movements	9 (11)	26 (32)	3 (11)	7 (16)	2 (18)	–	6 (16)	8 (11)	9 (19)	9 (19)
Purposeful movements	6 (8)	18 (12)	2 (8)	5 (18)	3 (17)	1 (9)	3 (8)	5 (13)	4 (9)	4 (9)
Muscle tension										
Relaxed	71 (88)	57 (70)	24 (89)	19 (70)	9 (82)	11 (100)	34 (89)	32 (84)	39 (85)	43 (93)
Tense	10 (12)	24 (30)	3 (11)	8 (30)	2 (18)	−	4 (11)	6 (16)	7 (15)	3 (7)
*Mechanically ventilated*										
Compliance with ventilator										
Tolerating ventilator	41 (51)	22 (27)	17 (63)	13 (48)	5 (45)	6 (55)	22 (58)	23 (61)	27 (59)	25 (54)
Activating alarms	–	17 (21)	–	4 (15)	–	–	1 (3)	–	–	1 (2)
Fighting ventilator	–	3 (4)	–	–	1 (9)	–	–	–	–	–
*Not mechanically ventilated*										
Vocalization										
Normal tone/no sound	38 (47)	25 (31)	9 (33)	5 (19)	4 (36)	4 (36)	14 (37)	13 (34)	18 (39)	16 (35)
Sighing/moaning	1 (1)	6 (7)	–	3 (11)	–	–	–	1 (3)	–	1 (2)
Verbal complaints	–	7 (9)	–	1 (4)	–	–	–	–	–	2 (4)
Tracheostomy^a^	1	1	1	1	1	1	1	1	1	1
CPOT-Neuro scores										
Median	0	3	0	3	1	0	0	1	0	0
Range	0–4	0–8	0–4	0–7	0–8	0–2	0–4	0–7	0–4	0–6
IQR	0–1	1–4	0−1	2−3	0–2	0–1	0–1	0–2	0–2	0–1.25
Mean (SD)	0.61 (1.04)	2.81 (1.91)	0.67 (1.11)	2.63 (1.62)	1.55 (2.29)	0.36 (0.67)	0.74 (1.11)	1.18 (1.47)	0.85 (1.26)	0.91 (1.94)

*IQR* interquartile range, *SD* standard deviation

^a^Patient with tracheostomy unable to vocalize and not mechanically ventilated

### Criterion validation

From those who were conscious, 52% could communicate but 19% of them screened positive for delirium and could not provide a reliable self-report of pain. CPOT-Neuro scores were compared with the pain intensity (Table 5) reported by these conscious patients who were negative on delirium screening. At rest pre-turning, 27 patients reported the presence of pain and 40 negated pain. Of these 67 patients, 56 could provide a score on the 0–10 FPT with a median pain intensity < 1. During turning, 51 patients self-reported the presence of pain compared to 26 who denied pain. Of these 77 patients, 62 could provide a numeric score with a median pain intensity > 3. At rest before other nociceptive procedures, 24 patients reported the presence of pain and 6 negated pain. From these 30 patients, 24 could provide a numeric score with a median pain intensity of 1. During other nociceptive procedures, 24 patients reported the presence of pain versus 6 who negated pain. Twenty-five patients could provide a numeric score with a median pain intensity of 4. Before the administration of an opioid dose, 8 patients reported the presence of pain while 4 denied pain (preemptive analgesia). Post-opioid administration, 5 patients reported pain and 4 did not. The median pain intensity decreased by one point post-opioid administration. Similar proportions of patients reported the presence of pain before (38%) and during NIBP (50%) as well as before (48%) and during soft touch (44%). Medians of pain intensity were 0 for most time points of non-nociceptive procedures, and was 1 during NIBP.

**Table 5:** Patients’ self-reports of pain intensity using the 0–10 Faces Pain Thermometer

Pain intensity	Procedures									
	Turning		Other nociceptive procedures		Opioid administration		Non-invasive blood pressure		Soft touch	
	Pre (*n* = 56)	During (*n* = 62)	Pre (*n* = 24)	During (*n* = 25)	Pre (*n* = 9)	Post (*n* = 9)	Pre (*n* = 47)	During (*n* = 47)	Pre (*n* = 58)	During (*n* = 57)
Median	0.50	3.25	1	4	6	5	0	1	0	0
Range	0–10	0–10	0–10	0–9	3–9	1–10	0–9	0–10	0–9	0–8
IQR	0–4	0–7	0–3	1–6.5	3–8	3–8	0–3	0–4	0–4	0–3
Mean	2.24	3.90	2.38	3.88	5.89	5.33	2.35	1.73	2.27	1.61
SD	2.28	3.37	3.28	2.93	2.37	3.08	2.72	2.72	2.77	2.33

*IQR* interquartile range, *SD* standard deviation

The CPOT-Neuro scores correlated moderately with self-reported pain intensity during turning (Spearman’s rho = 0.63, *p* < 0.001) and other nociceptive procedures (Spearman’s rho = 0.64, *p* = 0.001). At rest, CPOT-Neuro scores correlated significantly with self-reported pain intensity before turning (Spearman’s rho = 0.43, *p* = 0.001) and before other nociceptive procedures (Spearman’s rho = 0.42, *p* = 0.042).

### Ability of the CPOT-Neuro to classify patients with or without pain and at different levels of pain intensity during turning and other nociceptive procedures

The ROC curve analysis indicated an Area Under the Curve (AUC) of 0.76 (*p* < 0.001; 95% CI 0.65–0.87) for the presence versus absence of pain (yes/no) during turning (*n* = 77). Sensitivity was 77% and specificity was 69% corresponding to a CPOT-Neuro cut-off score ≥ 2. The same cut-off score was obtained for the presence of pain during other nociceptive procedures (*n* = 30) with an AUC of 0.84 (*p* = 0.011; 95% CI 0.63–1.00), and both sensitivity and specificity were 83%.

**Fig. 2:**
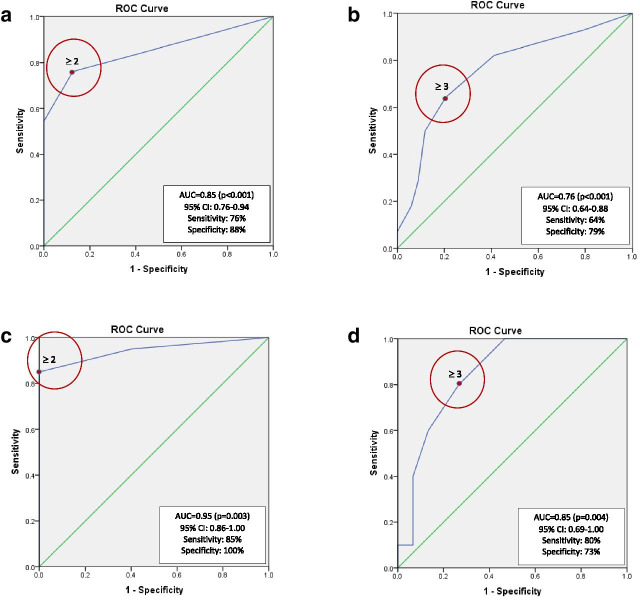
Receiver Operating Characteristic (ROC) curve analyses of CPOT-Neuro during turning (*n* = 62) and other procedures (*n* = 25) at different self-reported pain intensity criteria (≥ 1 and ≥ 5) using the 0–10 Faces Pain Thermometer. **a** Turning – Pain Intensity ≥1 **b** Turning – Pain Intensity ≥5 **c** Other nociceptive procedures – Pain Intensity ≥1 **d** Other nociceptive procedures – Pain Intensity ≥5. *AUC* area under the curve, *CI* confidence interval

ROC curve analyses were performed with pain intensity scores ≥ 1 and ≥ 5 used as reference criteria during turning and other nociceptive procedures and are displayed in Fig. 2. The ability of the CPOT-neuro to classify patients at these two pain intensity criteria varied from 76 to 95% for both turning and other procedures. The best CPOT-Neuro cut-off score that maximized sensitivity and specificity was ≥ 2 with all levels of pain intensity criterion ≥ 1, and was ≥ 3 with moderate to severe pain intensity criterion  ≥ 5, respectively.

### Discriminative validation

For the first data set, total CPOT-Neuro scores were significantly higher during turning compared to rest (Wilcoxon Signed Ranks test = − 11.17, *p* < 0.001), NIBP (Wilcoxon Signed Ranks test = − 9.93, *p* < 0.001) and soft touch (Wilcoxon Signed Ranks test = − 10.92, *p* < 0.001). After opioid administration, total CPOT-Neuro scores were significantly lower compared to before administration (Wilcoxon Signed Ranks test = − 2.97, *p* = 0.003). Total scores were similar during turning and other nociceptive procedures (Wilcoxon Signed Ranks test = − 0.42, *p* = 0.674). Total CPOT-Neuro scores were not significantly different across LOC, with medians of 3 in all 3 groups during turning and other nociceptive procedures (Kruskal Wallis test = 0.03 and 0.27, *p* = 0.986 and 0.265). Similarly, total CPOT-Neuro scores were not statistically different across sedation levels. Medians were 3 during turning and other nociceptive procedures for most groups except for a median of 2 in the awake group during turning (Kruskal Wallis test = 5.06 and 0.95, *p* = 0.080 and 0.624).

For the second data set, similar results were found, except for opioid administration, which could be assessed for only 11 patients (Wilcoxon Signed Ranks test = − 1.72, *p* = 0.086). Again, total CPOT-Neuro scores were not different across LOC, with medians of 3 for all 3 groups during turning (*n* = 81) (Kruskal Wallis test = 0.99, *p* = 0.611), and medians of 2.5 and 3 during other nociceptive procedures (*n* = 27)  (Mann–Whitney *U* test = 77.50, *p* = 0.697) in the altered LOC (*n* = 10) and conscious group (*n* = 17), respectively. In addition, total CPOT-Neuro scores during turning were not statistically different across sedation levels, with medians of 2 in the awake group (*n* = 18), 3 in the sedated group (*n* = 50), and 4 in the agitated group (*n* = 13) (Kruskal Wallis test = 3.53, *p* = 0.171). Similar results were found during other nociceptive procedures, with medians of 2 in the sedated group (*n* = 15), 3 in the awake group (*n* = 6), and 3.5 in the agitated group (*n* = 6) (Kruskal Wallis test = 0.37, *p* = 0.833).

### Interrater reliability

The interrater reliability of CPOT-Neuro scores between the research staff and ICU nurses was highest during turning with an ICC = 0.76 (95% confidence interval (CI): 0.68–0.82; *n* = 157). An ICC = 0.70 (95% CI: 0.50–0.83; *n* = 42) was found during other nociceptive procedures.

## Discussion

This is the first validation study of the French-Canadian and English versions of the CPOT-Neuro. The findings supported the validity and the interrater reliability of the tool. This validation was conducted among a heterogeneous sample of brain-injured ICU patients with various LOC and RASS scores ranging from − 4 to + 3. More than half of our study sample were conscious patients and 44% presented an altered LOC or unconsciousness.

Moderate positive correlations [46] were found between CPOT-Neuro and self-reported pain intensity scores at rest and during nociceptive procedures supporting criterion validation of the tool in conscious and non-delirious patients. Similar findings were found with the original version of the CPOT in medical, surgical, and trauma ICU patients [23]. Very few studies included brain-injured ICU patients able to self-report. In an Italian study of 300 observations from 50 brain-injured ICU patients, a lower correlation (Spearman rho = 0.38) was found between the original CPOT scores and self-reports of pain intensity at rest and during mobilization [14]. Similarly to the Italian study [14], a Spearman rho correlation of 0.44 between CPOT scores and self-reported pain intensity was found during 190 observations at rest and during turning of 66 trauma and neurosurgical ICU patients (50% with a brain injury) in a previous Canadian study [15]. In both of these studies, non-dependent data (i.e., multiple self-reports from the same patients) were used to calculate the coefficients which may inflate the correlation values.

The CPOT-Neuro could adequately classify patients who reported the presence of pain and various levels of pain intensity during nociceptive procedures. Overall, the performance of the CPOT-Neuro was better than reported for the original version of the CPOT in a previous study with brain-injured ICU patients (AUC = 0.72) [11]. The CPOT-Neuro cut-off score was higher when the reference criterion was set to moderate to severe pain intensity (≥ 5). In previous studies with the original version of the CPOT, cut-off scores varied from 2 to 3 to establish the presence of pain in the ICU population [23]. A cut-off score matching moderate to severe pain may be more useful in the ICU pain management decision making process to determine when opioids are most required. Replication studies with the CPOT-Neuro are necessary to confirm the cut-off score to be used.

The CPOT-Neuro was able to discriminate between nociceptive and non-nociceptive procedures as well as prior to and post-administration of an opioid dose which is clinically relevant. Indeed, we expect from a pain tool to discriminate between painful and non-painful conditions and to detect decreases in pain scores following opioid administration. Regarding the CPOT-Neuro items, it is worth mentioning that the frequency of a score of 2 for facial expression was higher in the CPOT-Neuro (36–49%) compared to original version of the CPOT (12–22%) during nociceptive procedures [10, 11]. Chookalayia and colleagues [47] highlighted issues with the items of body movements and muscle tension in the original version of the CPOT showing that they could not discriminate between the presence and the absence of pain in agitated ICU patients (*n* = 15). The modifications made in the development process of the CPOT-Neuro allowed a better representation of behavioral reactions exhibited by brain-injured ICU patients [17].

The CPOT-Neuro scores were similar across LOC groups and sedation levels during nociceptive procedures. This is a strength of this revised tool compared to the use of the original version of the CPOT in brain-injured ICU patients. Indeed, previous studies reported low CPOT scores (< 2) during nociceptive procedures in unconscious or deeply sedated brain-injured ICU patients [16, 20, 26] or with severe brain injury [12]. It is worth mentioning that nociception and pain are distinct but related concepts. Nociception is the neural process of encoding noxious stimuli, and pain is an unpleasant sensory and emotional personal experience associated with, or resembling that associated with, tissue damage [48]. Further, consciousness or memory/recall of events is necessary for the perception of pain. Previous studies report some individuals in a coma following a traumatic brain injury or a cardiac arrest could recall feeling pain when they were unconscious [49, 50]. We used nociceptive procedures known to be painful as reported by conscious ICU patients to validate the CPOT-Neuro in those with an altered LOC and unconsciousness [23]. Therefore, the behaviors included in the CPOT-Neuro can detect nociception which is likely to lead to pain. The International Association for the Study on Pain has acknowledged that the inability to communicate does not negate the possibility that an individual experiences pain [48]. In alignment with this statement and the evidence in the field, it appears reasonable to identify the CPOT-Neuro as a pain assessment tool.

Interrater reliability results were acceptable and met standards for reliability coefficients [51]. ICC was higher (> 0.80) between raters at the time of the training when exposure to the CPOT-Neuro was fresh. Only trained ICU nurses used the CPOT-Neuro for this validation study and for enrolled patients. Some of them could not use it shortly after their training and on a regular basis during the study period. Brief booster sessions of 15 min should be planned when enrollment becomes slow to review the use of the tool in order to maintain rating skills [52]. Indeed, many nurses who used the CPOT-Neuro in Canadian sites expressed their desire to get more training and exposure to the tool in their daily practice as they evaluated the tool to be easy and quick to use as well as clinically relevant [35].

In this study, we collected both CPOT-Neuro and self-reported pain scores for research purposes. The patient’s self-report of pain remains the reference standard measure of pain in those able to communicate in a reliable manner, and should be obtained whenever possible in clinical practice [53, 54]. Therefore, alternative pain assessment methods such as the CPOT and CPOT-Neuro should only be used when the patient’s self-report is unobtainable. Systematic pain assessments should be done on a regular basis using the most appropriate pain assessment method based on the patient’s ability to communicate [53, 54].

### Limitations

Several limitations must be addressed. Although we aimed for a heterogeneous sample of brain-injured ICU patients, those unconscious or agitated were less represented (< 20%) and further validation in these groups is required. It was not possible to blind raters to procedures leading to possible bias when scoring with the CPOT-Neuro during nociceptive procedures. However, the evaluation of interrater reliability allowed us to minimize this bias, and the data analyst was not involved in data collection. It was challenging to collect pain self-reports as more than half of conscious and awake patients had delirium or possible cognitive deficits related to their brain injury. Delirium can affect the capacity of an individual to communicate in a reliable manner, and self-reports of delirious patients were weakly (< 0.25) and non-significantly correlated to behavioral pain scores (with CPOT and BPS) in a previous study [55]. Therefore, the screening of delirium is important for the evaluation of criterion validation [23]. In this study, either the CAM-ICU or ICDSC was used for delirium screening; however, it was not measurable in a large proportion of patients due to deep sedation and/or coma [42]. Finally, because the CPOT-Neuro is based on the observation of behavioral reactions, it cannot be used in unresponsive patients (i.e., GCS of 3 or RASS of − 5).

## Conclusions

The French-Canadian and English versions of the CPOT-Neuro were found to be valid and reliable in this multi-site sample of brain-injured ICU patients at various LOC and levels of sedation. Indeed, the performance of the CPOT-Neuro appeared superior to the CPOT based on previous validation studies in brain-injured ICU patients. The feasibility of its use for validation purposes was described in a separate paper [35], but implementation studies are required to describe its feasibility in ICU daily practice.

## Supplementary Information

**Additional file 1.** Supplemental Material: French-Canadian and English versions of the CPOT-Neuro and Directives of Use

## Data Availability

The datasets generated and/or analyzed during the current study are not publicly available due to ethics guidelines but are available from the corresponding author on reasonable request.

## Abbreviations

APACHE: Acute Physiology and Chronic Health Evaluation
AUC: Area Under the Curve
CAM-ICU: Confusion Assessment Method for the Intensive Care Unit
CI: Confidence Interval
CPOT: Critical-Care Pain Observation Tool
GCS: Glasgow Coma Scale
ICC: Intraclass Correlation Coefficient
ICU: Intensive Care Unit
IQR: Interquartile Range
LOC: Level of Consciousness
NIBP: Non-Invasive Blood Pressure
RASS: Richmond Agitation-Sedation Scale
ROC: Receiver Operating Characteristic
TBI: Traumatic Brain Injury
