# Supplementary material for: Validation of the Critical-Care Pain Observation Tool-Neuro in brain-injured adults in the intensive care unit: a prospective cohort study
Source: Crit Care. 2021 Apr 13;25:142. doi: 10.1186/s13054-021-03561-1 (PMC8042624; doi:10.1186/s13054-021-03561-1)
Supplement: Supplementary file 1 — Additional file 1. Supplemental Material: French-Canadian and English versions of the CPOT-Neuro and Directives of Use [file 13054_2021_3561_MOESM1_ESM.docx]

Supplemental Material: French-Canadian and English versions of the CPOT-Neuro and Directives of Use

| **French-Canadian Version** | | | **English Version** | | |
| --- | --- | --- | --- | --- | --- |
| Item | Score | Description | Item | Score | Description |
| Expression Faciale | Détendue/neutre 0 | Aucune tension musculaire du visage | Facial Expression | Relaxed/neutral 0 | No facial muscle tension |
|  | Abaissement des sourcils 1 | Abaissement des sourcils |  | Brow lowering 1 | Lowering of the eyebrows |
|  | Contraction partie supérieure  visage ou grimace 2 | Abaissement des sourcils et tension de l’orbite (yeux serrés) ou grimace |  | Contraction of upper face or grimacing 2 | Brow lowering and eye tightening (wincing), grimacing |
| Larmes/  Rougissement visage | Absence 0 | Absence de ces réponses | Tearing/  Face Flushing | Absence 0 | Absence of both responses |
|  | Présence 1 | Présence de larmes et/ou de rougissement du visage |  | Presence 1 | Presence of tearing and/or face flushing |
| Mouvements Corporels | Absence de mouvements 0 | Immobile, ne bouge pas | Body Movements | Absence of movements 0 | Immobile, not moving |
|  | Mouvements non dirigés 1 | Mouvements lents, prudents, non dirigés vers site de douleur, flexion des membres |  | Non purposeful movements 1 | Non purposeful cautious/slow movements or limb flexion not aimed at reaching pain site |
|  | Mouvements de protection 2  et/ou agitation | Se dirige ou touche le site de douleur, les tubes ; mouvements de retrait ; agitation |  | Purposeful movements of 2  protection and/or restlessness/agitation | Trying to reach or touching/rubbing pain site, withdrawing and/or  pulling tube, attempting to sit up, thrashing, not following commands, striking at staff |
| Interaction avec Ventilateur | Tolère ventilation 0 | Alarmes non actives, se laisse ventiler | Compliance with Ventilator | Tolerating ventilator or movement 0 | Easy ventilation, no alarms |
|  | Active les alarmes 1 | Alarmes peuvent s’activer mais cessent spontanément |  | Activating alarms 1 | Alarms may be activated but stop spontaneously |
| **OU** | Combat ventilateur 2 | Bloque le ventilateur, déclenche constamment les alarmes | **OR** | Fighting ventilator 2 | Blocking ventilation, alarms frequently activated |
| Vocalisation | S’exprime normalement, 0  silencieux | Ton normal, demeure silencieux | Vocalization | Talking in normal tone 0  or no sound | Normal tone or no sound |
|  | Gémit, soupire 1 | Gémissements, soupirs |  | Sighing, moaning 1 | Sighing, moaning |
|  | Plaintes verbales, cris 2 | Plaintes verbales de dlr (aïe!); Cris |  | Verbal complaints, crying out 2 | Verbal complaints of pain (ouch!)  Crying out |
| Tension Musc. | Détendu(e) 0 | Pas de résistance aux mouvements passifs | Muscle Tension | Relaxed 0 | No resistance to passive movements |
|  | Tendu(e), rigide 1 | Résistance aux mouvements passifs |  | Tense, rigid 1 | Resistance to passive movements |
| **TOTAL** | **8** |  | **TOTAL** | **8** |  |

© Céline Gélinas To obtain permission of use, contact the author at [celine.gelinas@mcgill.ca](mailto:celine.gelinas@mcgill.ca)

##### Instructions d’utilisation du CPOT-Neuro

1. Le patient doit être évalué au repos pendant une minute pour obtenir une valeur de base sur l’échelle
2. Par la suite, le patient est évalué lors de procédures de soins (ex : mobilisation, succion endotrachéale, soins de plaie) afin de détecter tout changement de comportement pouvant être associé à la douleur.
3. Le patient doit être évalué avant l’administration d’un analgésique et au pic d’action de celui-ci afin de vérifier l’efficacité du traitement.
4. Le patient se voit toujours attribué le score le plus élevé sur chacun des énoncés de l’échelle pour un score total de 0 à 8.
5. La tension musculaire est évaluée en dernier lieu, surtout lorsque le patient est au repos puisque le fait d’effectuer des mouvements passifs (flexion et extension de l’avant-bras) peut déclencher d’autre réactions comportementales.

*Expression faciale et Réponses autonomiques*: Un patient présentant un visage détendu se voit attribuer un score de 0. L’observation de l’abaissement des sourcils donne un score de 1. Une contraction plus prononcée dans le haut du visage avec abaissement des sourcils et serrement des yeux équivaut à un score de 2. Un score de 2 est également attribué pour la grimace impliquant la contraction des muscles du haut et du bas du visage avec des yeux fermés serrés et contraction des muscles des joues (pli nasolabial). Le patient peut ouvrir la bouche ou mordre le tube endotrachéal s’il est intubé. L’infirmière porte également attention à la présence de larmes et/ou rougissement du visage (réponses autonomiques) pour un score de 1. En leur absence, un score de 0 est attribué.

*Mouvements corporels:* Un score de 0 est donné au patient qui est immobile. Lorsque des mouvements de flexion des membres ou des mouvements lents/prudents sont observés mais ne sont pas dirigés vers le site de douleur, un score de 1 est attribué. Lorsque les mouvements sont dirigés vers le site de douleur (par ex : site de la blessure, incision chirurgicale), que le patient tente de toucher à ses tubes, qu’il bouge sans cesse ou qu’il est agité, un score de 2 est donné.

*Interaction avec le ventilateur*: Pour le patient sous ventilation mécanique, l’infirmière prête attention à son interaction avec le ventilateur. Lorsque le patient se laisse ventiler sans déclencher les alarmes, un score de 0 est donné. Lorsque le patient déclenche les alarmes du ventilateur mais que celles-ci s’arrêtent spontanément, un score de 1 est attribué. Lorsque le patient combat le ventilateur et que l’infirmière doit intervenir (par ex : rassurer le patient, administrer un médicament), un score de 2 est alors donné. L’infirmière peut vérifier la position du tube endotrachéal ou la présence de sécrétions puisque ces facteurs peuvent influencer le score sans être associés à la douleur.

*Émission de sons*: Chez le patient non-intubé, l’infirmière porte attention aux sons émis par celui-ci. Un score de 0 est donné en l’absence de son émis ou lorsque le patient s’exprime dans un ton normal. Un score de 1 est donné lorsque le patient soupire ou gémit, et un score de 2 est donné lorsque le patient émet des plaintes ou des cris de douleur (Aïe! Ouch!).

*Tension musculaire*: Lorsque le patient est au repos, l’infirmière exécute une flexion et une extension du bras pour ressentir s’il y a résistance aux mouvements. L’infirmière peut ressentir si le patient résiste aux mouvements lors de la mobilisation au lit. Un score de 0 est donné lorsque le patient est détendu et peut être tourné sans résistance. Un score de 1 est attribué lorsqu’une résistance au mouvement est ressentie. Le patient est alors tendu ou rigide. Le patient peut également serrer les poings.

**Instructions about the use of the CPOT-Neuro**

1. The patient must be observed at rest for one minute to obtain a baseline value on the scale.
2. Then, the patient should be observed during standard care procedures known to be painful (e.g. turning, endotracheal suctioning, wound care) to detect any changes in the patient’s behaviors to pain.
3. The patient should be evaluated before and at the peak effect of an analgesic to assess the treatment efficacy.
4. The patient should be attributed the highest score observed for each item for a total score from 0 to 8.
5. Muscle tension should be evaluated last, especially when the patient is at rest because the stimulation of touch alone (when performing passive flexion and extension of the arm) may lead to behavioral reactions.

*Facial expression and Autonomic Responses*: A score of 0 is given when there is no muscle tension observable in the patient’s face. A score of 1 is given when lowering of the eyebrows is observed. A score of 2 refers to more pronounced tension in the upper face with eye tightening (or wincing) is observed in addition to brow lowering. A score of 2 is also given for a grimace which is a contraction of the full face including eyes tightly closed and contraction of the cheek muscles (nasolabial furrow). The patient may open their mouth, or if intubated, may bite the endotracheal tube. The nurse also pays attention to autonomic responses (i.e., tearing, face flushing) and gives a score of 1 in the presence of one or the other, or 0 when both of these responses are absent.

*Body movements*: A score of 0 is given when a patient is immobile. A score of 1 refers to non-purposeful movements such as limb flexion or slow/cautious movements not directed towards the pain site. When the movements are aimed at the pain site (e.g., injury, surgical incision) or the patient attempts to touch tubes, is thrashing or agitated, a score of 2 is given.

*Compliance with the ventilator*: Compliance with the ventilator is used when the patient is mechanically ventilated. A score of 0 refers to easy ventilation. The patient tolerates the ventilation and is not activating the alarms. A score of 1 is given when the patient triggers the alarms but they stop spontaneously. When the patient is fighting the ventilator and the nurse must intervene such as reassuring the patient or administering a medication, a score of 2 is given. The nurse may check for the position of the endotracheal tube or secretions as these factors may influence this item without being indicative of pain.

*Vocalization*: Vocalization is used in non-intubated patients able to vocalize. A score of 0 refers to the absence of sound or to the patient talking in a normal tone. A score of 1 is given when the patient is sighing or moaning, and a score of 2 for verbal complaints of pain (Aïe! Ouch!).

*Muscle tension*: When the patient is at rest, it is evaluated by performing a passive flexion and extension of the patient’s arm to feel any resistance to movements. The nurse can easily feel the patient’s resistance during turning in bed. A score of 0 is given when no resistance is felt during the passive movements or when the patient is turned in bed. A score of 1 refers to resistance during movements or turning. In other words, the patient is tense or rigid. The patient may also clench their fists.
